# Comparative Analysis of Open and Closed Nasal Fractures in Trauma Settings: Mechanisms, Intent, Surgical Interventions, and Outcomes

**DOI:** 10.3390/cmtr18010009

**Published:** 2025-01-22

**Authors:** Ahmad K. Alnemare

**Affiliations:** Otolaryngology Department, College of Medicine, Majmaah University, Al-Majmaah 11952, Saudi Arabia; a.alnemare@mu.edu.sa

**Keywords:** facial trauma, injury severity, nasal bone fracture, national trauma data bank, trauma mechanisms

## Abstract

Objectives: This study aimed to explore nasal fracture patterns, trauma mechanism and intent, treatment approaches, and mortality rates, offering insights for clinical practice and prevention in trauma settings. Design: This retrospective analysis was carried out using trauma data from the National Trauma Data Bank (NTDB) for the years 2013 to 2016. Main outcome measures: Trauma mechanism and mortality rates between closed and open fractures were conducted. Results: This study involved 122,574 closed and 9704 open nasal fractures to elucidate demographic, hospital, and clinical characteristics. Significant risk factors for open nasal fractures included a higher injury severity score, self-inflicted intent, unintentional causes, and firearm mechanism compared to assault injuries. Conclusions: Significant factors associated with open nasal fractures include injury severity, self-inflicted intent, trauma type, and firearm mechanisms, which notably increase the likelihood of open fractures. Findings highlight the need for targeted prevention, efficient resource allocation, and risk screening to enhance the management of complex facial traumas in the national trauma system.

## 1. Introduction

Nasal fractures represent some of the most common traumatic facial injuries treated in emergency departments and trauma centers. They often result from blunt force trauma to the nasal area, leading to displacement of the nasal bones [1,2,3]. While simple closed nasal fractures are routinely managed with closed reduction techniques, open nasal fractures are more complex injuries associated with laceration of overlying soft tissues and communication with the exterior nasal environments [4]. Thus, open nasal fractures raise concerns regarding bone instability or displacement, contamination, the need for surgical debridement, and complicated reconstructive demands, ultimately requiring multidisciplinary coordination among trauma surgery, otolaryngology, plastic surgery, and rehabilitation services.

Despite representing severe, resource-intensive traumatic injuries, there is limited evidence on the risk factors that predispose patients to open nasal fractures using robust national samples [5,6]. Most studies examine the epidemiology of nasal fractures in general, observing relationships with age, sex, and mechanism of injury based on relatively small case series from single institutions or regions. However, evidence of open nasal fracture subtypes is lacking [3,6,7]. Several trauma studies have noted associations between open nasal fractures and intentional self-harm mechanisms or penetrating trauma, but have not adjusted for potential confounders or identified other contributing factors in multivariate analyzes. Identifying vulnerabilities to these high-risk injuries can guide prevention initiatives, need assessments for reconstructive services, and allocate resources to trauma centers [7,8,9]. Pham et al. (2019) reported that the presence of an open wound to the nose is the most strongly associated injury for nasal fractures [10]. Characterizing these complex facial injuries is imperative for quality improvement in trauma care coordination, surgical management, and rehabilitation planning for this vulnerable patient population [6,11]. The clinical significance of this analysis extends beyond epidemiological description.

Using a large, nationally representative trauma database, this study aimed to investigate variations in injury mechanisms, intent, surgical interventions, and outcomes between open and closed nasal fractures. By identifying factors such as injury severity, self-inflicted intentionality, injury mechanism, vital signs, and outcome, we seek to bridge the gap between epidemiological data and clinical practice, ultimately enhancing the care provided to patients with these challenging injuries. The findings of this study can serve as a foundation for future prospective studies and help develop evidence-based interventions to improve outcomes in patients with open nasal fractures.

## 2. Methodology

### 2.1. Ethical Considerations

This analysis used de-identified data and received exemptions from the Institutional Review Board.

### 2.2. Data Source and Study Design

This retrospective study used the NTDB data from 2013 to 2016. Managed by the American College of Surgeons, the NTDB is the largest aggregated database of trauma patients in the United States and serves as a pivotal performance improvement tool for trauma care. This study followed STROBE guidelines.

### 2.3. Study Population and Variables

The NTDB was queried for adult patients with nasal fractures identified using the International Classification of Diseases, 9th Revision, Clinical Modification (ICD-9-CM) codes for both closed (802) and open (802.1) nasal bone fractures. Nasal fractures were classified as open when there was any breach in the overlying soft tissue that communicated with the fracture site. All other nasal fractures were classified as closed fractures. In cases with multiple nasal fracture components, the presence of an open component results in an open fracture. Baseline patient characteristics included age, sex, social history, and presence of hypotension (systolic blood pressure < 90 mmHg) upon admission. The severity of trauma was assessed using the Injury Severity Score (ISS) and Abbreviated Injury Scale (AIS) score for each body region. Comorbidities, mechanisms of injury, associated injuries, and specific facial fractures were also analyzed. Pan-facial fractures and open wounds on the nose and face were categorized and documented. Outcome measures included mean length of stay (LOS), intensive care unit (ICU) LOS, ventilator days, open or closed nasal reduction, complications, and mortality.

### 2.4. Statistical Analysis

Continuous variables were tested for normality using the Kolmogorov–Smirnov and Shapiro–Wilk tests. Categorical variables were assessed using frequency distributions. In the initial phase of the analysis, univariate logistic regression was used to identify the potential predictors of nasal fractures in patients with adult trauma. This was followed by multivariate logistic regression analysis, adjusting for continuous and categorical variables to determine independent predictors. Statistical significance was established with a two-sided *p*-value of less than 0.05. Missing data were handled using the default list-wise deletion method in Stata under the assumption that missingness was completely random (MCAR).

## 3. Results

### 3.1. Patient Demographics

**Figure 1:**
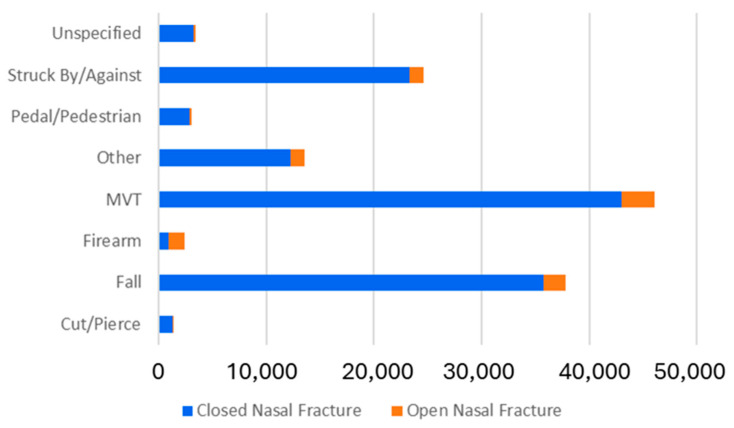
Trauma mechanism in open and closed nasal fracture (2013–2016).

In total, 133,278 patients with nasal fractures were identified, including 122,574 (92.0%) with closed nasal fractures and 9704 (8.0%) with open nasal fractures (Table 1). The median ages of the closed and open fractures were 43 and 41 years, respectively (*p* < 0.001). Overall, 71.9% were male, although open fractures had a higher proportion of males compared to closed fractures (74.2% vs. 71.8%, *p* < 0.001). The mechanism of injury differed significantly between the groups (*p* < 0.001), with closed fractures more likely to result from motor vehicle trauma (35.1% vs. 31.9%) or falls (29.2% vs. 21.7%), while open fractures most commonly resulted from firearms (14.5% vs. 0.8%, Figure 1). Most closed fractures were unintentional (76.9%), whereas a greater proportion of open fractures were self-inflicted (7.7% vs. 0.8%) or assault-related (19.7% vs. 21.1%).

### 3.2. Concomitant Facial Fractures

Concomitant facial fractures differed between closed and open nasal fractures (Table 2). Although all patients with open fractures had open nasal fractures, 8.4% also had evidence of closed nasal bone fractures. Patients with open nasal fractures had higher rates of both open (8.6% vs. 1.4%) and closed (3.1% vs. 6.6%) mandibular fractures. Similarly, a higher proportion of patients with open nasal fractures sustained open fractures of the malar/maxillary bones (12.8% vs. 0.5%), orbital floor (6.9% vs. 0.3%), and other facial bones (12.1% vs. 0.5%) compared to closed nasal fracture cases (all *p* < 0.001).

### 3.3. Vital Signs and Resuscitation Factors

Vital signs and resuscitation indicators also differed between the groups (Table 3). Patients with open nasal fractures had modestly higher rates of severe injury (ISS > 15; 32.7% vs. 28.0%), comatose status (GCS ≤ 8; 32.7% vs. 31.4%), tachycardia or bradycardia (48.5% vs. 46.5%), respiratory distress (29.5% vs. 28.5%), hypoxia (34.8% vs. 33.6%), and no signs of life (0.8% vs. 0.4%) than those with closed nasal fractures (all *p* < 0.05). However, the rate of hemodynamic instability was similar between groups. A lower proportion of patients with open fractures was transferred from other facilities (23.2% vs. 25.7%, *p* < 0.001).

### 3.4. Determinants of Open Nasal Fractures

Logistic regression analysis revealed the significant factors associated with the occurrence of open nasal fractures (Table 4). The fit of the model was statistically significant (LR chi^2^(14) = 5162.99, *p* < 0.0001), explaining 7.66% of the variance in the incidence of open nasal fractures (Pseudo R^2^ = 0.0766). The analysis indicated that the ISS was a notable predictor, with each unit increase in the ISS associated with a 0.4% increase in the odds of an open nasal fracture (OR = 1.004; 95% CI, 1.002–1.006; *p* = 0.001). Age had a marginal and non-significant influence on the likelihood of sustaining an open nasal fracture (OR, 1.000; 95% CI, 0.999–1.001; *p* = 0.254). Sex was not a significant determinant (OR = 0.999; 95% CI, 0.949–1.051; *p* = 0.970). In terms of injury intent, self-inflicted incidents significantly increased the odds of open nasal fractures (OR = 2.157; 95% CI, 1.855–2.509; *p* < 0.001), as did intentional incidents (OR = 2.166; 95% CI, 1.964–2.388; *p* < 0.001). The mechanism of injury also played a significant role, with incidents involving firearms markedly increasing the risk (OR = 8.839; 95% CI, 7.287–10.722; *p* < 0.001).

## 4. Discussion

Open nasal fractures are often part of more severe facial trauma, with a higher incidence of associated open fractures of the mandible, malar and maxillary bones, orbital floor, and other facial bones than closed nasal fractures [10,12]. The current analysis provides important information on the factors associated with open nasal fractures. In particular, open nasal fractures were more prevalent in a slightly younger demographic and had a higher incidence among men, suggesting age- and sex-specific patterns of nasal trauma. The data also revealed a significantly higher incidence of penetrating injuries in open nasal fractures, particularly firearm-related injuries, emphasizing the severity and violent nature of these incidents. Furthermore, self-inflicted injuries were markedly more common in open nasal fractures, pointing to different etiologies than closed fractures.

Patients with open nasal fractures showed a markedly higher incidence of associated open fractures of the mandible, malar and maxillary bones, orbital floor, and other facial bones than those with closed nasal fractures. This trend indicates that open nasal fractures are often associated with more severe facial trauma, underscoring the need for comprehensive evaluation and management of these cases. The contrast in the prevalence of these associated injuries highlights the varying degrees of trauma severity between closed and open nasal fractures, and underscores the importance of tailored treatment approaches for these patients [11,13]. Notably, a higher proportion of patients with open nasal fractures were severely injured (ISS > 15) and exhibited signs of severe trauma, such as higher rates of comatose state (GCS ≤ 8) and severe abnormalities in pulse rate, respiratory rate, and oxygen saturation. The data also showed a slightly higher percentage of patients with open nasal fractures arriving without signs of life and a marginally lower rate of inter-facility transfers, highlighting the urgent and critical nature of open nasal fracture cases in trauma settings [14,15]. These differences underscore the need for increased clinical vigilance and prompt and comprehensive management of patients presenting with open nasal fractures [15,16,17,18,19]. In terms of complications, there were slight but statistically significant differences in the rates of cardiovascular complications and mortality, with open nasal fractures exhibiting higher rates. This difference underscores the increased risk associated with open nasal fractures [15,17,20].

Consistent with previous literature, injury severity emerged as the strongest predictor in our regression model, with every 1-point increase in ISS conferring 0.4% higher adjusted odds of open nasal fracture [10,21]. Although the effect size appears modest, patients often present with a high ISS in the setting of penetrating trauma and polytrauma, amplifying this association. The significant impact of injury severity highlights the need for comprehensive trauma evaluations and coordinated care for patients at risk of multisystemic injuries, leading to adverse outcomes, such as facial fractures. Intentionality of the injury also plays an important role. Both self-inflicted and unintentional incidents more than doubled the odds of open nasal fractures compared with assault injuries. Self-inflicted open nasal fractures may result from gunshot wounds in attempted suicides, consistent with their strong overlap with the firearm mechanisms. Unintentional open nasal fractures have a variety of possible etiologies ranging from falls to motor vehicle collisions. Regardless of the intent, open nasal fractures signal high-energy transfer to the facial area and require prompt recognition and treatment.

Hwang et al. found that the predominant causes of injury included slips or falls (42.3%), violence (24.3%), sports-related incidents (19.2%), traffic accidents (8.9%), and work-related accidents (5.3%) [21]. This distribution was closely aligned with the data for closed nasal fractures. However, a notable deviation was observed in the context of open nasal fractures, in which firearm injuries were the most significant factor. Ballistic injuries often result in tissue damage, comminuted fractures, and contamination. Bullets and fragments can tear directly through the nasal soft tissue and bone or transmit explosive forces, leading to multisite fractures. These complex injuries require urgent surgical intervention and long-term reconstructive planning, particularly for concomitant brain and spinal cord injuries. Other sociodemographic factors, such as sex and obesity, did not significantly modify the odds of open nasal fractures.

This retrospective analysis had several limitations. The study used data from the NTDB in the US; therefore, the findings may not be directly applicable to other countries with different healthcare systems and injury patterns. The ICD-9 coding of injuries can involve inaccuracies or omissions that distort fracture classification. Key clinical variables related to fracture patterns, comminution, contamination, operative needs, reconstructive course, and patient-reported outcomes were not captured through registry data, thus constraining deeper insights relevant to head and neck trauma surgeons. A notable limitation of the NTDB dataset is its inability to distinguish between blunt trauma caused by firearms as objects and projectile wounds. This distinction could potentially affect the injury patterns and outcomes. Future trauma registries would benefit from incorporating this level of detail into the injury mechanism classification. More prospective studies with detailed mechanism classifications are needed to better understand the impact of specific firearm-related injury patterns on treatment approaches and outcomes. Furthermore, the NTDB data do not allow for long-term follow-up, which would provide a greater understanding of how open nasal fractures influence functionality, psychosocial well-being, and utilization of healthcare resources over time. Future studies should focus on collecting more detailed prospective data on nasal fracture treatment and outcomes to provide insights that are directly applicable to the practice of head and neck trauma surgeons. Additionally, the lack of patient-reported outcome measures (PROMs) limits our understanding of the long-term outcomes and quality-of-life implications of open and closed nasal fractures. Future prospective studies incorporating PROMs should address these knowledge gaps and provide a more comprehensive understanding of nasal fracture management.

Despite these limitations, our findings have significant implications for clinical decision-making in nasal fracture management. Our NTDB analysis provided practical insights into clinical practice and resource management. The data challenge traditional assumptions, showing that a significant portion of open nasal fractures were managed nonoperatively, which suggests that selective conservative management may be appropriate in certain cases. For resource allocation, the findings support maintaining integrated multidisciplinary teams, given the high rate of concomitant injuries, optimizing OR scheduling based on intervention timing patterns, and prioritizing resources by recognizing that most closed nasal fractures can be managed without immediate operative intervention. These insights will enable evidence-based protocols for efficient trauma care delivery while maintaining appropriate clinical outcomes. Otolaryngologists should adopt a tailored approach to managing open fractures, which may require more extensive surgical interventions, closer monitoring, and multidisciplinary rehabilitation. In contrast, closed fractures involve more operations of the nose, mouth, and pharynx. This study also highlights the importance of efficient resource allocation and targeted prevention strategies to minimize the burden of complex facial trauma on the healthcare system. Future prospective studies should compare the outcomes and decision-making strategies between open and closed nasal fracture management to provide guidance for ORL surgeons. The identification of factors associated with open nasal fractures underscores the need for risk screening and targeted preventive efforts to enhance trauma management. Although our analysis of the NTDB provides valuable insights into nasal fracture management within the U.S. trauma system, we acknowledge that healthcare delivery models vary significantly worldwide. The management patterns observed in our study reflect practices within a well-resourced trauma system with established protocols and readily available surgical specialists. However, these findings can inform practice in various global settings in several ways. In resource-limited environments, our data on conservative management outcomes could help guide decision-making when immediate surgical intervention is not available. Additionally, our findings regarding timings of intervention and resource utilization can assist international centers in optimizing their trauma protocols, recognizing that adaptation to local resources and healthcare infrastructure is essential. The high proportion of concomitant injuries in our cohort also highlights the importance of comprehensive trauma assessment regardless of geographic location.

## 5. Conclusions

This national analysis sheds light on vulnerability to sustaining open nasal fractures. ISS, self-inflicted injury intent, unintentional causes, and firearm-related mechanisms have emerged as the factors associated with open nasal fractures. Patients presenting with these high-risk characteristics should receive prompt and thorough evaluations for open nasal fractures, given their complex nature and need for urgent management. Targeting prevention initiatives for individuals at an elevated risk of self-harm or unintentional injuries involving firearms could meaningfully reduce the incidence of severe facial trauma. This study provides a framework to understand the epidemiology of open nasal fractures. However, further investigation is warranted on long-term patient outcomes, optimal treatment protocols, and the utilization of trauma center resources related to open nasal fracture care. Future research should also integrate hospital administrative databases with clinical outcomes to provide cost-effectiveness analyses and resource utilization patterns. This could be achieved through prospective multicenter studies incorporating both clinical and economic outcomes

## Figures and Tables

**Table 1:** Comparison of characteristics of closed and open nasal fractures.

Variable	Closed Nasal Fracture (*N* = 122,574)	Open Nasal Fracture (*N* = 9704)	*p*-Value
Age			<0.001
Median (Q1, Q3)	43.0 (26.0, 60.0)	41.0 (25.0, 58.0)	
Year of Injury			0.547
2013	31,983 (26.1%)	2520 (26.0%)	
2014	33,178 (27.1%)	2642 (27.2%)	
2015	33,833 (27.6%)	2628 (27.1%)	
2016	23,580 (19.2%)	1914 (19.7%)	
Age Group			<0.001
0–14	7744 (6.3%)	581 (6.0%)	
15–24	19,035 (15.5%)	1683 (17.3%)	
25–34	21,214 (17.3%)	1679 (17.3%)	
35–64	50,512 (41.2%)	4085 (42.1%)	
65+	24,069 (19.6%)	1676 (17.3%)	
Sex			<0.001
Male	87,969 (71.8%)	7202 (74.2%)	
Race			0.038
Black	15,574 (12.7%)	1275 (13.1%)	
Others	20,955 (17.1%)	1566 (16.1%)	
White	86,045 (70.2%)	6863 (70.7%)	
Mechanism			<0.001
Cut/Pierce	1235 (1.0%)	160 (1.6%)	
Fall	35,740 (29.2%)	2101 (21.7%)	
Firearm	948 (0.8%)	1410 (14.5%)	
MVT	42,999 (35.1%)	3097 (31.9%)	
Other	12,266 (10.0%)	1259 (13.0%)	
Pedal/Pedestrian	2853 (2.3%)	218 (2.2%)	
Struck By/Against	23,272 (19.0%)	1301 (13.4%)	
Unspecified	3261 (2.7%)	158 (1.6%)	
Intent			<0.001
Assault	25,874 (21.1%)	1860 (19.7%)	
Other	253 (0.2%)	37 (0.4%)	
Self-inflicted	909 (0.8%)	724 (7.7%)	
Undetermined	521 (0.4%)	72 (0.8%)	
Unintentional	91,939 (76.9%)	6730 (71.4%)	
Trauma Type			<0.001
Blunt	111,031 (92.9%)	7319 (77.7%)	
Burn	71 (0.1%)	7 (0.1%)	
Other/Unspecified	6208 (5.2%)	527 (5.6%)	
Penetrating	2186 (1.8%)	1570 (16.7%)	

Note: Age presented as median (Q1, Q3). Percentages are based on the total number of cases within each fracture category. MVT represents Motor Vehicle Traffic. Q1 and Q3 represent the first and third quartiles, respectively.

**Table 2:** Prevalence of other facial fracture types in closed vs. open nasal fractures.

Variable	Closed Nasal Fracture (*N* = 122,574)	Open Nasal Fracture (*N* = 9704)	*p*-Value
Closed Fracture of Mandible	8090 (6.6%)	303 (3.1%)	<0.001
Open Fracture of Mandible	1730 (1.4%)	838 (8.6%)	<0.001
Closed Fracture of Malar and Maxillary Bones	33,611 (27.4%)	2172 (22.4%)	<0.001
Open Fracture of Malar and Maxillary Bones	595 (0.5%)	1239 (12.8%)	<0.001
Closed Fracture of Orbital Floor	22,568 (18.4%)	1104 (11.4%)	<0.001
Open Fracture of Orbital Floor	349 (0.3%)	674 (6.9%)	<0.001
Closed Fracture of Other Facial Bones	29,109 (23.7%)	1526 (15.7%)	<0.001
Open Fracture of Other Facial Bones	590 (0.5%)	1173 (12.1%)	<0.001

Note: This table presents the distribution and comparison of different types of facial fractures in patients with closed versus open nasal fractures.

**Table 3:** Vital signs and clinical status of patients with nasal fractures.

Variable	Closed Nasal Fracture (*N* = 122,574)	Open Nasal Fracture (*N* = 9704)	*p*-Value
Severely Injured (ISS > 15)	34,362 (28.0%)	3172 (32.7%)	<0.001
Hemodynamically Unstable	27,122 (22.1%)	2211 (22.8%)	0.132
Comatose (GCS ≤ 8)	38,459 (31.4%)	3177 (32.7%)	0.005
Category of Pulse Rate (Severe if <60 or >100)	56,936 (46.5%)	4709 (48.5%)	<0.001
Category of Respiratory Rate (Severe if <12 or >25)	34,957 (28.5%)	2860 (29.5%)	0.045
Category of Oxygen Saturation (Severe if <92)	41,205 (33.6%)	3376 (34.8%)	0.019
Arrived with No Signs of Life	534 (0.4%)	80 (0.8%)	<0.001
Interfacility Transfer	31,489 (25.7%)	2256 (23.2%)	<0.001

Note: ISS, Injury Severity Score; GCS, Glasgow Coma Scale; the specific categories for pulse rate, respiratory rate, and oxygen saturation define the criteria for severe conditions. The percentages were based on the total number of cases in each category.

**Table 4:** Logistic regression analysis identifying factors associated with open nasal fractures.

Predictor	Odds Ratio (95% CI)	*p*-Value
ISS	1.004 (1.002, 1.006)	0.001
AGE	1.000 (0.999, 1.001)	0.254
GENDER (reference = Female)	0.999 (0.949, 1.051)	0.970
INTENT (reference = Assault)		
Other	1.156 (0.766, 1.744)	0.489
Self-inflicted	2.157 (1.855, 2.509)	<0.001
Undetermined	1.426 (1.050, 1.937)	0.023
Unintentional	2.166 (1.964, 2.388)	<0.001
Mechanism (reference = Cut/Pierce)		
Fall	0.235 (0.195, 0.285)	<0.001
Firearm	8.839 (7.287, 10.722)	<0.001
MVT	0.284 (0.235, 0.343)	<0.001
Other	0.469 (0.388, 0.568)	<0.001
Pedal/Pedestrian	0.305 (0.242, 0.384)	<0.001
Struck By/Against	0.423 (0.355, 0.504)	<0.001
Unspecified	0.352 (0.279, 0.445)	<0.001

Note: Reference categories are used for categorical variables to determine the relative risk compared to a baseline group. Statistical summaries included a likelihood ratio chi-square (LR chi^2^) of 5162.99 with a *p*-value < 0.0001, log likelihood of −31,139.275, and pseudo R-squared (R^2^) of 0.0766, based on an analysis of 128,919 cases.

## Data Availability

The datasets used and/or analyzed during the current study are available from the corresponding author upon reasonable request.

## Abbreviations

**Abbreviations**	**Definition**
AIS	Abbreviated Injury Scale
ICU	Intensive Care Unit
ISS	Injury Severity Score
LOS	Length of Stay
MCAR	Missing Completely At Random
NTDB	National Trauma Data Bank
GCS	Glasgow Coma Scale
ISSAIS	Injury Severity Score for Abbreviated Injury Scale
LR	Likelihood Ratio
STROBE	Strengthening the Reporting of Observational Studies in Epidemiology
US	United States

## References

[1] Hwang K., Ki S.J., Ko S.H. (2017). Etiology of nasal bone fractures. J. Craniofac. Surg..

[2] Erdmann D., Follmar K.E., Debruijn M., Bruno A.D., Jung S.-H., Edelman D., Mukundan S., Marcus J.R. (2008). A retrospective analysis of facial fracture etiologies. Ann. Plast. Surg..

[3] Andrades P., Pereira N., Rodriguez D., Borel C., Hernández R., Villalobos R. (2019). A Five-Year Retrospective Cohort Study Analyzing Factors Influencing Complications after Nasal Trauma. Cranial Maxillofac Trauma Reconstr..

[4] Ravikumar G., Sugapradha G.R. (2017). A study on faciomaxillary injuries in a tertiary care hospital. Int. Surg. J..

[5] Chan J., Most S.P. (2008). Diagnosis and management of nasal fractures. Oper. Tech. Otolaryngol.-Head Neck Surg..

[6] Hwang K., Yeom S.H., Hwang S.H. (2017). Complications of nasal bone fractures. J. Craniofac. Surg..

[7] Chou C., Chen C.-W., Wu Y.-C., Chen K.-K., Lee S.-S. (2015). Refinement treatment of nasal bone fracture: A 6-year study of 329 patients. Asian J. Surg..

[8] Allred L.J., Crantford J.C., Reynolds M.F., David L.R. (2015). Analysis of pediatric maxillofacial fractures requiring operative treatment: Characteristics, management, and outcomes. J. Craniofac. Surg..

[9] Imahara S.D., Hopper R.A., Wang J., Rivara F.P., Klein M.B. (2008). Patterns and outcomes of pediatric facial fractures in the United States: A survey of the National Trauma Data Bank. J. Am. Coll. Surg..

[10] Pham T.T., Lester E., Grigorian A., Roditi R.E., Nahmias J.T. (2019). National analysis of risk factors for nasal fractures and associated injuries in trauma. Craniomaxillofac. Trauma Reconstr..

[11] Kraft A., Abermann E., Stigler R., Zsifkovits C., Pedross F., Kloss F., Gassner R. (2012). Craniomaxillofacial trauma: Synopsis of 14,654 cases with 35,129 injuries in 15 years. Craniomaxillofac. Trauma Reconstr..

[12] Tochigi K., Miyashita K., Aoki S., Sakamoto H., Omura K., Tanaka Y. (2023). Characteristics of nasal foreign bodies and equipment on complications during removal procedures. Laryngoscope.

[13] Davari R., Pirzadeh A., Sattari F. (2023). Etiology and epidemiology of nasal bone fractures in patients referred to the otorhinolaryngology section, 2019. Int. Arch. Otorhinolaryngol..

[14] Katz A., Alerte E., Akhavan A., Kuruvilla A., Ibelli T., Liu H., Etigunta S., Taub P.J. (2022). Can frailty indices predict surgical risk in open reduction and fixation of facial fractures?. J. Craniofac. Surg..

[15] Duane T.M., Sercy E., Banton K.L., Blackwood B., Hamilton D., Hentzen A., Hatch M., Akinola K., Gordon J., Bar-Or D. (2022). Factors associated with delays in medical and surgical open facial fracture management. Trauma Surg. Acute Care Open.

[16] Renner G.J. (1991). Management of nasal fractures. Otolaryngol. Clin. North Am..

[17] Wang W., Lee T., Kohlert S., Kadakia S., Ducic Y. (2019). Nasal fractures: The role of primary reduction and secondary revision. Facial Plast. Surg..

[18] Sanders T., Rhodes H., Boscia J., Biswas S. (2023). Predictors of facial fractures in trauma patients: Retrospective review at a level I trauma center. Am. Surg..

[19] Canas M., Fonseca R., Diaz L., De Filippis A., Afzal H., Aldana J.A., Machica C., Leonard J., Liang S.Y., Bochicchio K. (2023). Open Mandible and Maxillary Fractures Associated with Higher Risk of Infection in Victims of Assault. Surg Infect (Larchmt).

[20] Chouinard A.-F., Troulis M.J., Lahey E.T. (2016). The acute management of facial fractures. Curr. Trauma Rep..

[21] Hwang K., Yoon J.M. (2023). Analysis of Nasal Bone Fractures: A 17-year Study of 3785 Patients. J. Craniofac. Surg..

